# Frequency of Fabry disease in a juvenile idiopathic arthritis cohort

**DOI:** 10.1186/s12969-021-00563-9

**Published:** 2021-06-12

**Authors:** Luciana Paim-Marques, Amanda Virginia Cavalcante, Islane Verçosa, Paula Carneiro, Marcia Souto-Maior, Erlane Marques, Simone Appenzeller

**Affiliations:** 1 Medical Physiopathology Program, School of Medical Science, University of Campinas, São Paulo Campinas, Brazil; 2 Pediatric Rheumatology Unit, Albert Sabin Children’s Hospital, Fortaleza, Ceará Brazil; 3 Division of Immunology & Rheumatology, Department of Pediatrics, University of Florida, Gainesville, FL USA; 4 Pediatrics Department, Public Health School of Ceará, Fortaleza, Ceará Brazil; 5 Ophthalmology Department, CAVIVER Institute, Fortaleza, Ceará Brasil; 6 Ophthalmology Unit, General Hospital of Fortaleza, Fortaleza, Ceará Brasil; 7 College of Medicine, University of Fortaleza (UNIFOR), Fortaleza, Ceará Brazil; 8 Genetics Division, Albert Sabin Children’s Hospital, Fortaleza, Ceará Brazil; 9 Pediatrics Department, Unichristus University, Fortaleza, Ceará Brazil; 10 Rheumatology Unit, School of Medical Sciences and University of Campinas (UNICAMP), Campinas, São Paulo Brazil; 11 Autoimmune Laboratory- School of Medical Science, University of Campinas, Campinas, São Paulo Brazil

**Keywords:** Fabry disease, Juvenile arthritis, Chronic arthritis, Alpha-galactosidase a, GLA variants

## Abstract

**Background:**

Fabry disease (FD) is a rare, X-linked, multisystemic lysosomal storage disorder (LSD) that results from a deficiency in the hydrolase alpha-galactosidase A (⍺-GalA). During childhood, classic FD symptomatology is rare. The majority of children may show non-specific symptoms, including in the musculoskeletal system. The prevalence of FD among juvenile idiopathic arthritis (JIA) patients is unknown.

**Objective:**

This study aimed to identify the frequency of FD in a JIA cohort, characterizing early clinical symptoms, enzyme titers, and *GLA* genotyping.

**Methods:**

Children with JIA followed in a tertiary Children Hospital cohort were selected. Clinical, laboratory and familiar information were recorded. Molecular genetic testing to detect *GLA* gene mutations was performed in girls and enzymatic analysis in boys.

**Results:**

In 89 patients (56.2% female, age at disease onset: 8.93 ± 4.35 years), one male (1.12%) patient presented pathogenic mutation in *GLA* gene, *c.1244 T > C p.L415P*, one female patient had a variant of uncertain significance *c.38C > T (p.Ala13Val).* Three additional (3.4%) patients had the enzymatic activity of alpha-galactosidase slightly decreased. We observed the presence of intronic variants in 44.44% of patients in our cohort: *c.1000-22C > T*; *c.370-81_-77del*; *c.640-16A > G*; *c.10C > T*; *c.548-125C > G* and *c.-12G > A.* These variants and their combination were associated with clinical symptoms in our cohort.

**Conclusions:**

The incidence of FD in our cohort was 1.12%. Intronic variants were associated with symptoms previously described in the literature. Screening for FD in JIA may be a reasonable strategy for those with an atypical pattern of pain.

## Background

Fabry disease (FD) is a rare, X-linked, multisystemic lysosomal storage disorder (LSD) that results from a deficiency in the hydrolase alpha-galactosidase A (⍺-GalA) caused by a *GLA* gene mutation. Its birth prevalence is estimated at 1:40.000–170.000 [1]. Neonatal screening has recently found a higher FD incidence: 1:3.100 in Italy and 1:1.500 among males in Taiwan [2, 3].

FD, a recessive X-linked disease, affects predominately male patients. The female carrier may present milder symptoms if X inactivation is present. These differences influence the diagnostic methods, clinical signs, and life expectancies. Male patients can be screened with enzyme titers, while female patients should have a genetic test done directly.

The ⍺-GalA deficiency in patient lysosomes with FD causes a progressive accumulation of the glycosphingolipid globotriaosylceramide (Gb3) in cells of many organ systems resulting in a chronic inflammatory process [4]. FD should be suspected in individuals presenting acroparesthesias or other classic manifestations such as angiokeratomas, gastrointestinal symptoms, exercise intolerance, ocular abnormalities (*cornea verticillata*), decreased sweating, renal and cardiac involvement. Central nervous system presentation may include transient ischemic attacks and strokes predominantly in the vertebrobasilar system. However, in early childhood, FD may present with mild non-specific symptoms frequently affecting the musculoskeletal system. Peripheral neuropathic pain, fever, arthritis, and elevated erythrocyte sedimentation rate (ESR) can be observed [5–8].

High disease suspicion is necessary at the early stages of the disease, and screening in high-risk patients is a cost-effective strategy for identifying FD patients [9]. Musculoskeletal features are frequently observed in FD. On the other hand, juvenile idiopathic arthritis (JIA) is the most frequent chronic inflammatory arthritis disorder detected in childhood. This study aimed to identify FD frequency in a JIA cohort by characterizing early clinical symptoms, enzyme titers, and *GLA* genotyping.

## Materials and methods

Consecutive JIA patients classified according to ILAR criteria [9, 10] followed in the pediatric rheumatology outpatient clinic at Albert Sabin Children’s Hospital were invited to participate in this cross-sectional study from December 2014 to December 2017.

The local ethics committee approved this study (Albert Sabin Childhood Hospital, Fortaleza, Ceará, Brazil, **CAAE:** 37270414.0.0000.5042), and all patients and their legal representatives, if children under 18, have signed the informed consent and assent form.

### JIA history

We obtained demographic and disease characteristics through a careful chart review for each patient, such as age, sex, age at disease-onset, JIA subtype, articular, and extra-articular manifestations. *Immunologic tests were rheumatoid factor (RF) by latex agglutination test, human leukocyte antigen (HLA) B27 by polymerase chain reaction (PCR), and antinuclear antibodies (ANA) by indirect immunofluorescence assay (IIFA) on Human epithelial type 2 (HEp-2 cells). Per protocol, a positive ANA required an anti- ds DNA, Smith, RNP, SSa, SSb, and anti-cardiolipin antibodies by enzyme-linked immunosorbent assay (ELISA).*

### Study questionnaire

JIA patients were inquired about FD features through a structured questionnaire based on early signs and symptoms applied by the treating physician [11]. This questionnaire contained queries about clinical symptoms (heat intolerance, hypo/hyperhidrosis, chronic fatigue, abdominal distension, dyspepsia, diarrhea, gastric fullness sensation, weight gain difficulty, tinnitus, dysacusis, acroparesthesia), physical exam findings (telangiectasia, angiokeratoma), past medical history (stroke and transient ischemic attack), and family history (stroke, transient stroke, sudden death, end-stage renal or FD).

### Clinical evaluation

All patients were evaluated by a board-certified pediatric rheumatologist who performed a complete clinical, osteoarticular, and neurological exam at study entry.

JIA patients underwent a thorough eye examination by a board-certified pediatric ophthalmologist, and a second board-certified specialist reviewed the positive findings. Refractive errors were measured by a hand-held auto-refractor keratometer retinomax K plus 2. The anterior segment (cornea, iris, and lens crystalline) was evaluated by slit-lamp examination. The optic nerve, macula, and posterior pole vessels were analyzed with direct ophthalmoscopy. A tear breakup time (TBUT) test was performed after placing a drop of fluorescein in the cul-de-sac to determine keratoconjunctivitis. A board-certified pediatric ophthalmologist evaluated the presence of cornea verticillata during eye evaluation.

The patients were also evaluated through a transthoracic 2D echocardiogram (Echo) and a twelve-channel electrocardiogram (ECG) to analyze conduction disturbance performed by a board-certified pediatric cardiologist.

A study of serum creatinine, 24-hour urinary microalbuminuria, and urinary sodium assessed kidney involvement.

### Genetic testing

The genetic test was sponsored by Shire Brasil and carried out in the outpatient clinic by a trained nurse. Blood was drawn after patients and legal representatives’ re-authorization and placed at five blood spots on filter paper duly identified with the patient’s, doctor’s, and nurse’s data.

For males, an initial screening of the *⍺-GalA enzyme and the acidic sphingomyelinase (control) enzyme activity and measurement of globotriaosylsphingosine (lyso-Gb3) by high-performance liquid chromatography (HPLC) was performed by tandem mass spectrometry at Centogene (Germany) with normal limits above 3,1* μmol*/l/h, and below 1.8 ng/ml, respectively.*

For women or male patients with abnormal enzyme activity, the GLA gene analysis (ref: NM_000169.2) was conducted by PCR and sequencing of the entire coding region and highly conserved exon-intron splice junctions. This test has been developed and validated by Centogene AG for clinical purposes. Patients with genetic abnormalities or ⍺-GalA enzyme below average values had their first and second-degree relatives (parents, grandparents, and siblings), when possible, screened for FD with an appropriate genetic investigation (enzyme levels or gene identification), and referred to genetic monitoring.

The variants were described according to the ACMG classification [12]. ACMG recommended the following modifiers: Pathogenic, likely pathogenic, uncertain significance, likely benign, or benign [12].

### Statistical analyses

All statistical analyses were performed using SPSS 20.0 software package. Results are shown in absolute number and percentage or mean and standard deviation (SD). Chi-square or Fischer exact test was used to compare categorical variables. The continuous variables were compared by analysis of variance (ANOVA). A *p*-value ≤0.05 was considered clinically significant.

## Results

A total of 89 JIA patients (mean age of 15.80 ± 3.95) were included, and we observed a majority of females (56.17%) and oligoarticular JIA subgroup (47.2%). The mean age of disease onset was 8.93 ± 4.35 years. The subtype classification, clinic, laboratory abnormalities of JIA patients were summarized in Tables 1 and 2. We did not observe significant differences in patient demographics and clinical characteristics among those enrolled in the study and those who did not agree to participate (data not shown).

**Table 1:** Frequency of subtypes, clinical, laboratorial and drugs used in JIA patients

Features	N (%)
JIA subtypes:	
Oligoarticular	42 (47.20)
Negative RF Polyarticular	17 (19.10)
Enthesitis related	15 (16.90)
Systemic	10 (11.20)
Positive RF Polyarticular	3 (3.36)
Psoriatic	1 (1.12)
Undifferentiated	1 (1.12)
Symptoms:	
Acroparesthesia	47 (52.80)
Difficulty gaining weight	30 (33.70)
Heat Intolerance	26 (29.50)
Hyperhidrosis	22 (24.70)
Dyspepsia	19 (21.30)
Tinnitus	18 (20.20)
Peripheric Neuropathy	17 (19.10)
Abdominal distention	14 (15.70)
Chronic fatigue	14 (15.70)
Diarrhea	11 (12.40)
Dysacusis	8 (9.00)
Gastric fullness sensation	6 (6.74)
Angiokeratoma	2 (2.24)
Telangiectasia	00
Family History:	
Stroke	38 (42.70)
Sudden death	22 (24.71)
kidney failure	11 (12.40)
Transient attack	2 (2.24)
Fabry	1 (1.12)
Laboratory:	
ANA	13 (14.60)
Microalbuminuria (66 patients)	9 (12.32)
HLA-B27	6 (6.74)
Rheumatoid Factor	3 (3.39)

**Table 2:** Cardiac Abnormalities in JIA Cohort and subtype descriptions - 74 patients – 20 cardiac abnormalities occurrence in 17 (22.90%) patients

Features	Total N (%)	Presentation x JIA Subtype
Right Bundle Branch Block		4 polyarticular
	10 (52.63)	1 systemic
		3 oligoarticular
		2 Entesitis-related
Mitral Valvar Prolapse		1 polyarticular
	4 (20)	2 oligoarticular
Ventricular Hypertrophy		1 polyarticular -LV (conc.)
	3 (15.78)	1 systemic - RV
		1 Oligoarticular + FD - LV (conc.)
Valvar Regurgitation:		Mitral: 1 Oligoarticular + FD
	3 (15.78)	Pulmonary: 1 Systemic
		Tricuspid: 1 Systemic

Legends: *LV* Left Ventricle, *RV* Right ventricle, *Conc* concentric. The mitral regurgitation was a pathologic insufficiency with LV hypertrophy, while pulmonary and tricuspid regurgitations were physiologic

The genetic tests were performed in all (56.17%) female patients, while enzyme activity was performed in 39 (43.82%) males of our cohort. The results identified 4 (4.49%) males with decreased enzyme activity. For that reason, we had a total of 54 (60.67%) patients with genetic tests done.

One of 89 (1.12%) patients (male, an admixture of Caucasian and Native South American, oligoarticular JIA with positive ANA with no previous treatment) presented diminished ⍺-GalA values (0,4 μmol/l/h) and abnormal lyso-Gb3 levels (67,8 ng/ml). His genetic test showed the *GLA* variant *c.1244 T > C p.L415P* (Ref: Serebrinsky, 2006) confirming FD [13]. That patient presented with hands and feet burning pain (acroparesthesia) at 5 years old, associated with a frequent low-grade fever after exercising or sun exposure. He also reported significant anhidrosis, fatigue, abdominal pain, and diarrhea. At the age of eleven, he reported bilateral ankle pain and swelling. He consulted by Pediatric Rheumatology for evaluation. His physical exam and workup showed angiokeratomas around the belly bottom, cardiac abnormalities, and *cornea verticillata*. The chronic ankle arthritis was clinically observed; however, the other complaints were uncommon in the JIA set of symptoms. His pedigree was rich for strokes, heart attack, and transient ischemic attack in family members under age 50. This patient also had intronic GLA variants, c.370-81_-77del (rs5903184) on intron 2, c.640-16A > G (rs2071397) on intron 4, c.1000-22C > T (rs2071228) on intron 6 e, c.-10C > T (rs2071225) in region 5’UTR exon 1. Once he started the enzyme replacement, his symptoms improved: hypohidrosis, abdominal pain, dyspepsia, heat intolerance, acroparesthesias, and angiokeratomas. However, no changes in the *cornea verticillata* and arthritis was observed. Magnetic resonance imaging of the right ankle showed tibiotalar, and tibiotarsal edema with synovial thickening. Methotrexate (15 mg/m^2^/week) was added to his therapeutic plan with subsequent joint swelling resolution.

Another female oligoarticular JIA patient presented a previously unreported heterozygous variant in exon 1 of the *GLA* gene *c.38C > T p.Ala13Val* (No reference). This variant of uncertain significance is located in a non-conserved nucleotide and a frankly conserved amino acid position, with a physical-chemical difference between the amino acid alanine and valine (Alamut v.2.4). Polyphen-2, SIFT, and MutationTaster analysis predict this variant as likely benign, but acroparesthesias, weight gain difficulties, and a *cornea verticillata* was observed. The patient also presented the following intronic GLA variants, c.370-81_-77del (rs5903184) on intron 2, c.640-16A > G (rs2071397) on intron 4, c.1000-22C > T (rs2071228) at intron 6 e, c.-10C > T (rs2071225) in region 5’UTR exon1. Although she denied any FD symptoms, her mother presented an early stroke but a negative initial genetic test.

A third oligoarticular JIA female with acroparesthesia complaints presented a heterozygous variant on the *GLA gene, on* exon 1. The *c.48 T > G* p.Leu16Leu (rs201449986) is considered benign (by Online Mendelian Inheritance in Man (OMIM), Clinvar and HGNC (HUGO Gene Nomenclature Committee) due to the change of the same codons, and because it is not in a splicing sequence. The statistical association of this variant with FD clinical symptoms, laboratory and *GLA* variants are described in Table 3.

**Table 3:** Statistics correlation (*p*-value) between genetic variants and CIHs and clinical signs of Fabry disease significant *p* < 0.05

Symptoms	c.1000-22C > T	c.370-81_-77del	c.640–16 A > G	c.-10 C > T	*c1244 T > C*	*c.38 C > T*	Hap1	Hap2	Hap3
AlfaGal Abn.	0.624	1.000	1.000	0.429	0.073	0.927	1.000	1.000	1.000
GB3 Abn	1.000	0.501	0.501	0.240	0.036	0.964	0.448	0.448	1.000
Heat Intolerance	**0.012**	**0.022**	**0.022**	0.116	0.091	0.091	0.098	0.098	0.325
fatigue	0.756	1.000	1.000	0.531	0.236	0.764	0.719	0.719	1.000
Tinnitus	0.202	1.000	1.000	0.531	0.236	0.764	0.719	0.719	0.234
Dysacusis	0.643	1.000	1.000	0.492	0.909	1.000	1.000	1.000	0.325
Acroparesthesias	0.278	0.775	0.775	0.437	1.000	0.473	0.764	0.764	0.613
Hyperhidrosis	0.222	0.102	0.102	0.624	0.273	0.273	0.493	0.493	1.000
Abdominal Dist	0.745	0.730	0.730	0.519	0.218	0.782	1.000	1.000	1.000
Dyspepsia	1.000	0.478	0.478	0.571	0.200	0.800	0.709	0.709	1.000
Diarrhea	0.718	0.212	0.212	0.733	0.145	0.145	0.405	0.405	1.000
Weight Gain Diff	0.775	0.213	0.213	***0.024***	0.309	0.691	0.322	0.322	1.000
Angiokeratoma	0.436	0.291	0.291	0.127	***0.018***	0.982	0.255	0.255	1.000
Valvar Abn	0.132	0.419	0.419	0.308	0.186	0.814	0.217	0.217	***0.031***
Arrhythmias	1.000	0.602	0.602	0.465	1.000	0.907	0.572	0.572	1.000
Visual Changes	0.073	***0.046***	**0.046**	***0.005***	0.213	0.787	***0.01***	***0.01***	0.521
*Corneal Changes*	**0.002**	***0.003***	***0.003***	***0.012***	0.255	0.401	***0.05***	***0.05***	1.000
*Cornea Vertic*	0.070	0.208	0.208	0,292	0.064	0.064	1.000	1.000	0.183
*Cataract*	0.426	1.000	1.000	0.894	1.000	0.979	1.000	1.000	0.064
*Ant. Chamb Ch*	***0.004***	***0.006***	***0.006***	***0.011***	1.000	0.128	0.164	0.164	0.343
Stroke FH	0.184	0.565	0.565	0.645	0.436	0.436	0.756	0.756	0.307
Trans. attack FH	0.186	0.081	0.081	0.240	***0.036***	***0.001***	0.448	0.448	1.000
Sudden death FH	1.000	0.346	0.346	0.586	0.327	0.673	0.510	0.510	1.000
kidney failure FH	0.686	0.660	0.660	0.423	1.000	0.891	1.000	1.000	0.379
Fabry FH	0.436	0.291	0.291	0.127	***0.018***	0.982	0.255	0.255	1.000

Legend: **Hap 1** = *c.*-10C > T, c.370-77_-81del, c.640-16A > G, c.1000-22C > T, **Hap 2**- c.370-77_-81del, c.640-16A > G, c.1000-22C > T, **Hap 3** = c.548-125C > G, c.1000-22C > T, c.-12G > A. Abn: abnormal, *Dist* Distention, *Diff* difficulty, *cornea Vertic* cornea verticillata, *Ant. Chamb Ch* Anterior chamber changes,, *FH* Family history

The enzymatic activity of ⍺-GalA has a standard range > 3.1 μmol/l/h. We found a slight decreased in 3 (3.4%) additional patients with a total of four (4,49%) abnormal enzymatic essays. Two patients had history and clinical symptoms suggestive of FD (hypohidrosis, acroparesthesia and dyspepsia, weight gain difficulty, familial history of sudden death, and end-stage renal disease). The third had no family history suggestive of FD. All three patients had repeated negative genetic tests for FD.

Furthermore, we identified a total of 18 (22.90%) patients with cardiac abnormalities, 10 (52.63%) patients had right bundle branch block, 4 (20%) presented mitral valve prolapse, 3 (15.78%) had valvar regurgitation. We also observed 3 (15.78%) patients with ventricular hypertrophy; one systemic JIA patient with right ventricular hypertrophy secondary to pulmonary hypertension, one polyarticular JIA patient with left ventricular hypertrophy, and our index patient with a left concentric ventricular hypertrophy.

A total of 54 (60.67%) patients had genetic testing. Twenty-six of 54 (48.14%) had *GLA* variants (intronic and exonic). The overwhelming majority of these patients (92.30%) presented multiple intronic *GLA* variants (Table 4). 24 (92.30%) of the 26 showed *c.1000-22C > T* (rs2071228) on intron 6, 16 (61.53%) with variant *c.370-81_-77del* (rs5903184) on intron 2, 15 (57.69%) patients with variant *c.640-16A > G* (rs2071397) on intron 4; 8 (30.76%) of them with *c.10C > T* (rs2071225) on 5’UTR exon 1; 7 (26.92%) patients with variant c.548-125C > G (rs2071396) on intron 3, and 4 with c.-12G > A (rs3027585) on 5’UTR exon 1.

**Table 4:** *GLA* variants observed in JIA Patients cohort and its frequency in Normal population according to Vep Ensembl

Variants	Description	*GLA* Location	Vep Ensembl Freq.
*c.-10C > T*	rs2071225	5’UTR of exon 1	0.09546
*c.-12G > A*	rs3027585	5’UTR of exon 1	0.06110
*c.370-81_-77del*	rs5903184	2	0.1571
*c.548-125C > G*	rs2071396	3	0.1372
*c.548-162A > T*	Never described	3	Never described
*c.640-16A > G*	rs2071397	4	0.1447
*c.1000-22C > T*	rs2071228	6	0.2542

We also observed presence of complex intronic haplotypes (CIH) in 44.44% of the total tests performed. The intronic variants as well as the CIH had positive correlations with FD symptoms (Table 3). They were grouped as **Haplotype 1**- *c.-10C > T, c.370-77_-81del, c.640-16A > G, c.1000-22C > T* in 8 (14.81%), **Haplotype 2**- *c.370-77_-81del, c.640-16A > G, c.1000-22C > T* in 7 (12.97%), **Haplotype 3**- *c.548-125C > G, c.1000-22C > T, c.-12G > A* in 4 (7.40%),; **Haplotype 4**- *c.548-125C > G, c.1000-22C > T* in 2 (3.70%), **Haplotype 5**- *c.370-81_-77del, c.548-125C > G, c.640-16A > G, c.1000-22C > T in 1(1,85%)* patient*.*

## Discussion

We observed a frequency of 1.12% of FD in our cohort. There are no epidemiological studies of FD in Brazil. However, the frequency expected for live male births in the general population is 0.0025% (*p* = 0.0088) [14]. In the pediatric population, a Portuguese group studied a cohort of 292 patients with JIA and its association with FD. However, they did not find a classic pathogenic mutation [9].

The ethnicity of the affected child [admixture of Caucasian and Native South American (Indian)] is not included in the populations of higher incidence for FD [2, 3].

In this cohort, we found three exonic *GLA* variants. The first variant, *c.1244 T > C,* was in our index case described as pathogenic and referenced by Serebrinsky et al. in 2006 as disease-causing according to ACMG variant classification recommendations [12].

The second variant (*c.38C > T p.Ala13Val*) has not been described before, and it is located in a non-conserved nucleotide and weakly conserved amino acid position, with small physicochemical differences between the amino acids alanine and valine (Alamut v.2.4). Software analyses by Polyphen-2, SIFT, MutationTaster, and Align-GVGD predict this variant as probably benign according to ACMG recommendations of interpretation of sequence variations [12].

The third variant in *GLA* exon 1 *c.48 T > G* (rs201449986) was considered likely benign [12]. This abnormal sequence does not alter an amino acid residue and is not located within the splice consensus sequence, according to Sequence Project (http://evs.washington.ed/EVS/;). This allele frequency is 1/6728.

Patients with classic FD have no residual or around 30-35% of ⍺-GalA enzyme activity [15]. For diagnosis, an increased level of Gb3 in lysosomes is required [16]. Its inheritance is X-linked and recessive, which means that the female heterozygous genotype presents incomplete penetrance due to X inactivation. The mildest disease allows women to have residual enzyme activity; for that reason, the genetic analysis is the gold standard for diagnosis [17]. The enzymatic activity can be measured in peripheral blood cells or dry blood spots. In our study, all analysis was done with dry blood spots. The enzymatic activity is very variable among FD patients and different organs [18]. These variations are a challenge to establish thresholds of FD pathogenicity [18].

In this study, we identified 4 (4.5%) male patients with decreased α-GalA activity levels. One confirmed the diagnosis of FD, while three others had negative genetic tests. The decreased α-GalA levels could be caused by a pre-analytical alteration of the samples (humidity, inadequate drying of the blood spots after collection, or extensive *GLA* duplication or deep-intronic mutation not well detected by the current method. Those patients had a second confirmatory negative genetic test.

A defective ⍺-GalA leads to accumulation of undegraded substrates [globotriaosylceramide and globotriaosylsphingosine (Gb3 and Lyso Gb3)] inside lysosomes, acting as damage-associated molecular patterns (DAMPs) or stimulating DAMP production. This production activates an inflammatory pathway, inducing apoptosis and a toll-like receptor- 4 (TLR4) mediated innate immune system pro-inflammatory cytokines secretion (IL-1β and TNF-α) [19]. These different cellular mechanisms contribute to the different phenotypic expression of FD [20]. These cytokines secretion are a characteristic trace of autoinflammatory disorders [21]. The recognition of Gb3 or lyso-Gb3 as antigens also influences the invariant natural killer T cell (iNKTs) to induce the release of other inflammatory cytokines such as interferon-gamma (IFN-γ), tumor necrosis factor-alpha (TNF-α), interleukins (IL): IL-4, IL-5, IL-9, IL10, IL13, and IL-17. This inflammatory cascade produces a continuous stimulus responsible for the induction and maintenance of the autoimmune response. The activation of the above-mentioned inflammatory pathway explains the presence of autoimmune and autoinflammatory features in FD. Beyond the classic FD symptoms, our index patient had positive ANA and RNP antibodies and chronic oligoarthritis (bilateral ankles).

The high frequency of cardiac involvement in our cohort (Table 2) is probably related to JIA, which can involve all cardiac structures, including pericardium, myocardium, endocardium; coronary vessels; valves, and conduction system [22]. However, FD also causes cardiac abnormalities, including conduction abnormalities, valvular dysfunction, arrhythmias in childhood, evolving to ventricle concentric hypertrophy in non-treated patients [23]. Our index patient had mitral valve prolapse with reflux and left ventricular concentric hypertrophy, cardiac manifestations frequently observed in FD.

The main musculoskeletal symptoms described in early FD is acroparesthesia. However, chronic inflammatory joint and bone diseases (polyarticular, oligo and monoarticular, gout, osteoporosis), degenerative joint conditions, neurologic arthropathy (Charcot’s foot) [7], Heberden-like nodules [24]*,* and also myositis have been described [8]. Nowadays, the coexistence of FD and autoimmune disease has gained increased visibility in the medical literature, and patients with FD and systemic lupus erythematosus [25, 26], rheumatoid arthritis [27], autoimmune hypothyroidism [28], Ig A nephropathy [29], and granulomatosis with polyangiitis [30] have also been described. Patients with FD and rheumatic manifestations have a significant delay in FD diagnosis that can last up to 16 years or more [31]. The most common associated mutations observed in FD patients presenting with rheumatic manifestations were R118C and A143T [31].

**Fig. 1:**
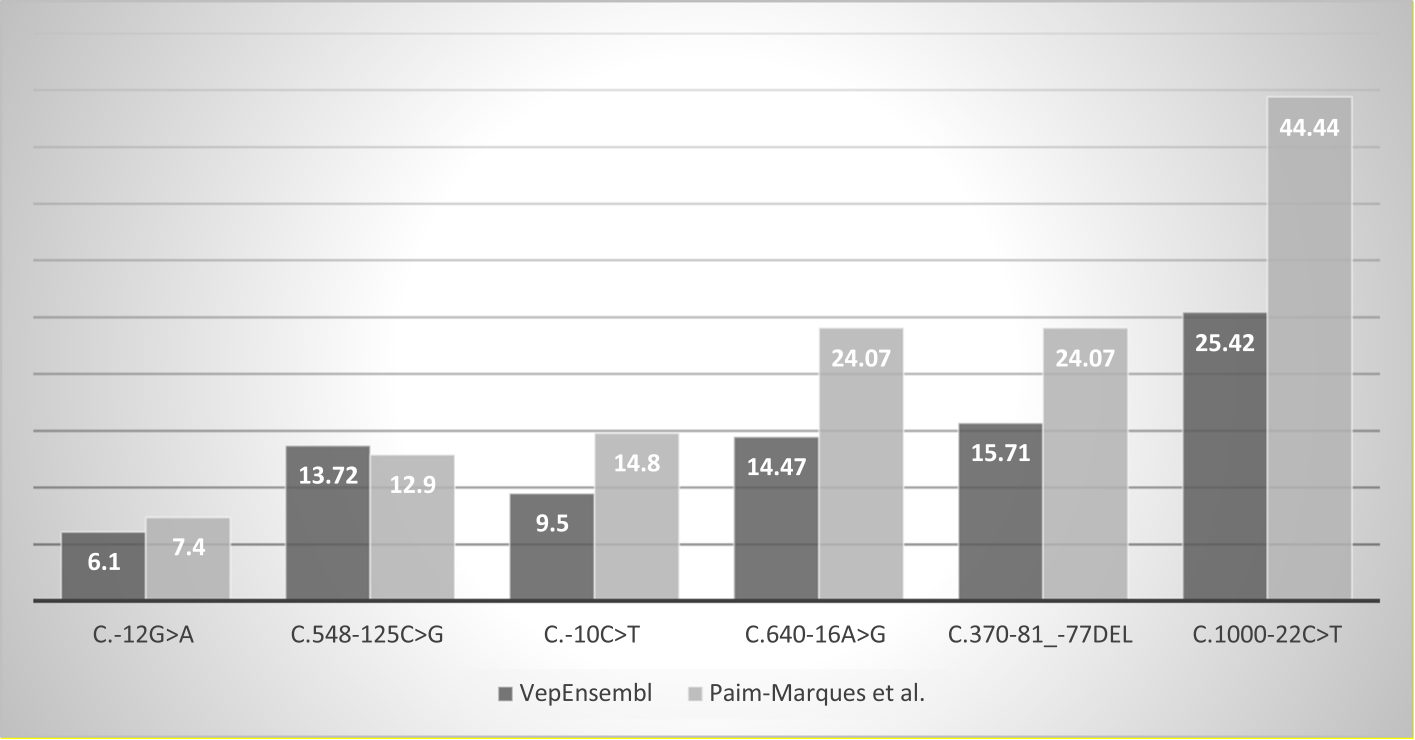
Frequency of Intronic *GLA* variants in the averagenormal population (according to Vep Ensembl) compared to JIA patients’ cohort

Another interesting finding in our study was the presence of *GLA* intronic mutations in patients with JIA. Six intronic variants were identified (Table 4), and a comparison between Vep Ensembl data on the normal population, and our cohort suggested an increased frequency in some variants (Fig. 1). Three single nucleotide polymorphisms (SNP) [*c.370-81_-77del* (rs5903184), *c.-12G > A* (rs3027585) and *c.-10C > T* (rs2071225)] are relatively common to different ethnic groups, with a frequency of the minor allele about 10% in the British population [32], and 12% in Latin populations (OMIM). These SNPs are common to the Portuguese population, the Brazilian citizens’ greatest ancestor [33]. Those variants were found in 12 (22.22%) *c.370-81_-77del*, 8 (14.81%) *c.-10C > T* and 4 (7.40%) *c.-12G > A* in the 54 tested patients.

As the previous variants, other SNP and its combination, denominated CIH, have been described as associated with Fabry-similar symptoms.

The two SNP of the α-GalA gene *c.1000-22C > T* [rs2071228] and *c.640-16A > G* (rs2071397) were associated with the presence of angiokeratomas and acroparesthesias in patients with hypertrophic cardiomyopathies without FD [34]. In vitro and in vivo analyses have shown that polymorphisms in the 5’UTR region can alter the α-GalA gene expression, with possible clinical relevance, particularly in male patients with *GLA* variants associated with a high reduction in enzyme activity [35]. The *c.-10 T* allele, found in 15% of this study’s positive results, was previously associated with a decrease in α-GalA activity in leukocytes [33]. It has a possible correlation with neurological injuries such as stroke, transient ischemic attack, white matter injury, fine fiber neuropathy in patients with peripheral neuropathy [36], and patients with FD [35]. In our study, this variant was associated with the difficulty of weight gain and ocular changes. Classic ocular manifestations in FD are observed by the age of 4, while heterozygotes present it later, around age 10 [37].

The *c.-10 T* allele, located in the 5′ non-coding region, has been associated with a decrease in the expression of α-GalA [34], altering in the promoter gene the nuclear protein binding site [38]. Studies are still needed to determine the real role of this variant in *GLA*. Recent data suggested that reduced enzyme activity, even with standard α-GalA levels, may be a risk factor in Parkinson’s disease [39]. There are numerous descriptions of the *c.-10 T* allele and Fabry-simile manifestations [34].

The most frequent intronic variant in our cohort was *c.1000-22C > T* (rs2071228), observed in 24 (44.44%) of 54 patients with increased frequency when compared to the normal population (25.42%) (Table 4, Fig. 1). This variant, located in intron 6, is phenotypically associated with FD and idiopathic hypertrophic cardiomyopathy by the bank Vep Ensembl. It is also associated with some CIH that seem to translate enzymatic alteration with glycosphingolipids’ accumulation [38]. Haplotypes are a combination of inherited alleles at adjacent *loci*. There are numerous reports of groups of alleles causing Fabry simile changes and FD per se. Gervas-Arruga et al., studied a ICH (*c.-10C > T, c.369 + 990C > A, c.370-81_370-77delCAGCC, c.640-16A > G, c.1000-22C > T*) in the *GLA* gene. They evaluated the enzymatic levels in cells (fibroblasts and leukocytes) in the plasma and the enzyme’s quantitative expression. The results suggested an altered expression pattern of the studied gene, without sufficient abnormality of enzyme levels in plasma, leukocytes, and skin fibroblasts to cause FD. However, glycosphingolipids accumulation in fibroblasts, renal, and glomerular tubular cells have been described [38].

Another study described a similar CIH on *GLA* in a patient with FD’s early systemic onset. This patient carried only the haplotype (*−10C > T, c.370-77_-81del, c.640-16A > G*, *c.1000-22C > T)*, suggesting that those variants located in a promoter and the intronic regulatory region could cause disease even without the presence of exonic abnormalities [40]. In our cohort, we had 8 (14.8%) of the 54 tests that presented this same haplotype (*−10C > T, c.370-77_-81del, c.640-16A > G, c.1000-22C > T*), including the patient with FD and the patient with *c.38C > T variant* who presented *cornea verticillata*. Haplotypes 1 and 2 were associated with visual changes and corneal abnormalities, and haplotype 3 had a positive association with valve changes (Table 1).

There was no association of acroparesthesias/peripheral neuropathies with ICHs, despite their incidence in half of our sample. A limited number of genetic tests in our cohort may have influenced the possible positive associations between the variants found and reported clinical signs.

In our JIA cohort, we observed a variety of clinical symptoms related to FD. Almost 50% described acroparesthesias, and a third of the patients had weight gain difficulty, while 42% had a family history of stroke. All these features could be associated with chronic arthritis and its treatment. Our index patient was initially treated with enzyme replacement, considering FD was misdiagnosed as JIA. Despite a significant improvement of anhidrosis, muscular, abdominal pain, and GB3 levels, he had persistent ankle swelling with synovial thickening, suggesting JIA’s coexistence. Methotrexate significantly improved his symptoms. We found another patient with a variant of uncertain significance (VOUS) for FD. Unfortunately, young age is an obstacle for the identification of FD or FD-like symptoms. These patients need surveillance with continued follow-up and laboratory evaluation, to determine if these variants will cause future damages, especially the females.

We observed FD as a comorbidity in 1.12% of our JIA cohort, but the small number of JIA patients in this cohort was a limitation for this study. We only included 50% of our cohort, mostly due to logistic issues (missing appointments, incomplete clinical evaluation).

Another limitation relates to the genetic test. Unfortunately, we were not able to offer it for all patients. Although we can assume that patients with normal enzyme levels do not present exonic pathogenic changes, we cannot conclude the same for intronic variants and *GLA* haplotypes. We also had no access to patient’s family medical records. Therefore, we could not explain the high incidence of familiar history of vascular events and confirm other possible confounders such as diabetes, obesity, or antiphospholipid syndrome.

## Conclusion

In our cohort, FD was present in 1.12% of JIA patients. FD can present autoimmune features, and a high index of suspicion is necessary for the diagnosis. Pediatric rheumatologists should be aware that FD could present with similar classic autoimmune disease features, but they can also co-exist. We need to be careful with those patients with JIA and “dysautonomia” symptoms (persistent extremities or diffuse pain, gastroparesis or abdominal pain, absence of sweat, and arrhythmias). With more thorough family history, searching for strokes, sudden death and heart attacks at a young age (< 50), and kidney disease leading to transplant. Those questions are crucial for FD diagnosis, and early disease identification is an effective strategy to avoid kidney transplants and premature death with the enzyme replacement [9]. In the future, we hope to perform enzyme activity and genetic tests in all suspected patients.

## Data Availability

All data generated or analyzed during this study are included in this published article [and its supplementary information files].

## Abbreviations

⍺-GalA: Alpha-galactosidase A
ANA: Antinuclear antibodies
ANOVA: Analysis of variance
CIH: Complex intronic haplotypes
DAMPs: Damage-associated molecular patterns
ds DNA: Double-stranded deoxyribonucleic acid
ECG: Electrocardiogram
Echo: Echocardiogram
ELISA: Enzyme-linked immunosorbent assay
ESR: Erythrocyte sedimentation rate
FD: Fabry disease
Gb3: Globotriaosylceramide
HEp-2: Human epithelial type 2
HGNC: HUGO gene nomenclature committee
HLA: Human leukocyte antigen
HPLC: High-performance liquid chromatography
IFN-γ: Interferon-gamma
IIFA: Immunofluorescence assay
IL: Interleukins
ILAR: International league against rheumatism
IOP: Intraocular pressure
JIA: Juvenile idiopathic arthritis
LSD: Lysosomal storage disorder
Lyso-Gb3: Globotriaosylsphingosine
OMIM: Online mendelian inheritance in man
PCR: Polymerase chain reaction
RF: Rheumatoid factor
RNP: Ribonucleoprotein
SNP: Single nucleotide polymorphism
TBUT: Tear breakup time
TLR4: Toll-like receptor- 4
TNF-α: Tumor necrosis factor-alpha
VOUS: Variant of uncertain significance
